# Environmental disruptors and testicular cancer

**DOI:** 10.1007/s12020-022-03171-z

**Published:** 2022-08-29

**Authors:** Fabiana Faja, Sandro Esteves, Francesco Pallotti, Gaia Cicolani, Silvia Di Chiano, Enrico Delli Paoli, Andrea Lenzi, Francesco Lombardo, Donatella Paoli

**Affiliations:** 1 Laboratory of Seminology—Sperm Bank “Loredana Gandini”, Department of Experimental Medicine, “Sapienza” Università di Roma, Rome, Italy; 2 Andrology and Human Reproduction Clinic, Av. Dr. Heitor Penteado, 1464, Campinas, Brazil; 3 Faculty of Health, Department of Clinical Medicine, Aarhus University, Aarhus, Denmark

**Keywords:** Environmental endocrine disruptors, Testicular cancer, Male reproductive health, Environmental pollution

## Abstract

**Purpose:**

Testicular cancer (TC) is the most common malignancy among young adult males. The etiology is multifactorial, and both environmental and genetic factors play an essential role in the origin and development of this tumor. In particular, exposure to environmental endocrine disruptors (EEDs), resulting from industrialization and urbanization, seems crucial both in pre-and postnatal life. However, the lack of long-term studies on a wide caseload and the difficulty in evaluating their toxic effects in vivo make it challenging to establish a causal link. This review aims to discuss the main human epidemiological studies currently available in the literature to define a possible association between these chemicals and TC.

**Methods:**

A comprehensive Medline/PubMed and Embase search was performed, selecting all relevant, peer-reviewed papers in English published from 2002 to January 2022. Other relevant papers were selected from the reference lists.

**Results:**

To date, literature evidence is limited due to the scarcity and heterogeneity of human studies and shows controversial data, highlighting the complexity of the topic. However, most human epidemiological studies seem to point toward a correlation between EEDs exposure and TC.

**Conclusion:**

Although the molecular mechanisms are not yet fully understood, the role of EEDs in TC onset is plausible, but several factors, such as the individual genetic background, the exposure time, and the complex mechanism of action of these chemicals, do not allow defining the causal link with certainty and make further studies necessary to investigate this complex topic.

## Introduction

Environmental endocrine disruptors (EEDs) are ubiquitous substances that mimic estrogenic and androgenic action disturbing the physiological hormonal homeostasis.

These elements often result from human activity deriving from industrialization and urbanization [1]. EEDs, whose exposure in postnatal life occurs via food, skin, and air, include a heterogeneous group of substances such as pesticides, solvents, chemicals used in plastic production, personal care products, clothes, and hydraulic and electronic devices [2]. Exposure can also occur in prenatal or early postnatal life through the placenta and breastfeeding [3]. Some EEDs are resistant to degradation and can persist in the ecosystem for a long time, constituting an environmental pollution problem [4]. Given the importance of hormonal mechanisms for proper sexual development, the action of a substance that impairs this physiological homeostasis could lead to reproductive consequences. Although further studies are needed, EEDs might affect puberty timing in postnatal life and impair testis development in prenatal life [5, 6]. These reproductive disorders are defined as testicular dysgenesis syndrome (TDS) and comprise different clinical conditions, among which hypospadias, cryptorchidism, infertility, and low testosterone levels [7].

Another pathology included in TDS is testicular cancer (TC), which represents the most common solid malignancy in men of reproductive age and whose incidence has increased over the last 50 years. In particular, this variation of incidence has been described to be relatively mild in countries with an already high incidence (virtually 0% in countries from northern Europe) up to 2–4% average annual growth in countries with a relatively low TC incidence (central and eastern Europe, Latin America, etc.) [8, 9].

Even if the etiology is multifactorial, epidemiological studies suggest that some clinical conditions appear to promote the development of TC and the main risk factors are cryptorchidism [10] and a family history of TC [11]. Familiarity for TC suggests the presence of genetic contribution to the development of these neoplasms as demonstrated by the discovery of specific polymorphisms reported in the literature, such as those affecting *BAK1*, *DMRT1*, *TERT-CLPTM1L*, *KITLG*, androgen receptor gene, and *PDE11A* [12–14].

Although scientific evidence is controversial, EEDs could represent one of the putative causes of testicular carcinogenesis. Animal studies highlighted severe toxic effects on male reproductive health, but the lack of long-term studies on a wide caseload makes it difficult to evaluate any association between EEDs and TC in humans [6]. Furthermore, other factors also contribute to making this link unclear, such as the gap between the time of EEDs exposure and the manifestation of effects, the combined result of several EEDs with different modes of action, and the individual genetic background that affects the response to a specific EED and the absence of standardized clinical protocols applied in human studies [15]. However, EEDs exposure in prenatal life seems to be associated with an increased risk of TDS, underlying their detrimental effect on the reproductive health of newborns [16, 17].

Therefore, despite the complexity of this topic, this review aims to briefly illustrate the mechanisms by which EEDs impair the hormone balance and describe those that may play a role in the development of TC, discussing the main human epidemiological studies currently available in the literature.

## Methods

A comprehensive Medline/PubMed and Embase search was performed using the following keywords “endocrine disruptors”, “environmental pollutants”, “testicular cancer”, “male urogenital diseases”, “pollution”, “phthalates” (or “phthalates esters”), “polychlorinated biphenyls” (or “PCBs”), “pesticides”. Relevant, peer-reviewed papers in English published from 2002 to January 2022 have been considered. Other relevant papers were selected from the reference lists.

## Effects of EEDs

The disruption of the physiological hormonal homeostasis by EEDs can occur through several mechanisms, such as altering hormone levels, inhibiting or stimulating their production and metabolism, or changing their transport through the body [18].

Since most of them are structurally similar to endogenous hormones, EEDs were initially thought to act as imperfect ligands of nuclear hormone receptors able to modify their signaling [19]. However, evidence has demonstrated that they can impair hormonal balance, interacting with nonsteroid receptors, transcriptional coactivators, and enzymatic pathways involved in steroid biosynthesis and/or metabolism [20]. Moreover, epidemiological and animal studies showed that EEDs could exert harmful effects on the exposed individual and successive generations modifying epigenetic patterns and suggesting a transgenerational inheritance [21–23].

Like hormones, EEDs can act at low doses impacting more than higher doses on the target tissue, which can be the liver, kidney, thyroid gland, or testis. It should be emphasized that understanding their mechanisms of action represents a challenge due to several factors, such as their non-traditional dose-response curve and their different effect depending on the age of exposure. Furthermore, while during adulthood EEDs toxicity is caused by higher concentrations, during development also low dose exposure can exert a detrimental effect for a long time [24].

The impact of EEDs exposure on the onset of TC is still under debate, but clinical evidence suggests that these chemicals can disrupt testicular function, assuming an association with urogenital diseases [25].

The increase in the incidence of TC observed in recent decades has led to the assumption that it could be correlated with the exposure to several pollutants resulting from industrialization and urbanization. Nevertheless, the studies currently available in the literature show discordant data highlighting the complexity of the topic.

EEDs comprise a large heterogeneous class of chemicals used commercially for different purposes, but in this review, we will describe only those that may play a role in testicular carcinogenesis.

## Main EEDs correlated to TC risk in literature

### Phthalates (Ps)

Ps, considered the main EEDs, are widespread in industrial processes and commercial goods. Ps are inexpensive synthetic chemicals commonly used as plasticizers in packaging, personal care products, industrial plastics, medical devices, and pharmaceuticals. Due to the lack of covalent bonds, exposure to heat over time can promote their esters’ migration into food [26].

In vitro studies demonstrate that phthalate esters may interact with androgen and estrogen receptors acting as estrogenic and antiandrogenic compounds [27].

Moreover, in vitro and in vivo toxicology studies show that Ps and their metabolites may be associated with congenital abnormalities (cryptorchidism, hypospadias), semen quality impairment, testicular germ cell cancer [25, 28–30], confirming the role of these chemicals in disrupting the hormonal balance.

Furthermore, Ps interfere with male reproductive system development impairing the function of Leydig and Sertoli cells [25]. Literature evidence in both human and animal models show that all these alterations may correspond to a clinical condition known as TDS, from which TC can arise [31, 32].

### Polychlorinated biphenyls (PCBs)

PCBs are persistent organic pollutants recognized as causing adverse effects on humans and ecosystems. Before their production ended in 1993, these chemicals were produced commercially for decades for several applications, such as adding polymeric building materials as plasticizers or flame retardants. Further, PCBs were used as a dielectric fluid in capacitors in fluorescent lighting ballasts. Their high persistence and ability to bioaccumulate along the food chain have made PCBs ubiquitous environmental contaminants.

PCBs include more than 200 possible congeners that exhibit a variety of chlorine substitution patterns and that have been classified into three groups based on structural, biological, and pharmacokinetic properties: estrogenic/neurotoxic (Group 1); antiestrogenic and immunotoxic, dioxin-like (Group 2); enzyme-inducing, phenobarbital (PB)-type cytochrome P450 inducers (Group 3) [33].

Although an association between PCBs and TC has been proposed [34–37], a causal relationship still remains to be clarified, leaving this topic open to further research.

### Pesticides

The effect of pesticides and insecticides has also been examined in relation to the male reproductive system. The literature shows that occupational exposure could impair semen quality, reducing seminal volume, percentage of motility and increasing seminal pH, abnormal sperm head morphology, and leukocyte concentration [36, 38–40]. Moreover, pesticides interfere with sex hormone concentrations decreasing serum luteinizing hormone and testosterone levels [39].

Dichloro-diphenyl-dichloroethylene (p,p’-DDE*)* represents one of the main pesticides investigated for the association with TC in literature [34, 35, 41–44]. p,p’-DDE, which can accumulate in adipose tissue and is poorly excreted, is a metabolite of dichloro-diphenyl-trichloroethane and a potent androgen receptor antagonist [45], commonly used as a pesticide until it was banned in the 1970s–1980s [46].

Another organochlorine pesticide widely distributed in the environment is hexachlorobenzene (HCB). HCB is a fungicide with physical properties, such as vapor pressure and water solubility, that make it persistent in the ecosystem and able to bioaccumulate. As p,p’-DDE, HCB has also been investigated in relation to TC, but literature evidence points to controversial data [35, 36, 41–44].

Finally, TC has also been associated with exposure to chlordane and its derivates (oxychlordane, trans-nonachlor, cis-nonachlor) [35, 41, 43], organic pollutants used as pesticides, and then prohibited in many countries due to their toxicity and persistence. Exposure can occur mainly through ingestion of contaminated food, inhalational or dermal routes and they can be stored for a long time in the body due to their high lipophilicity [47].

## Epidemiological studies

**Fig. 1:**
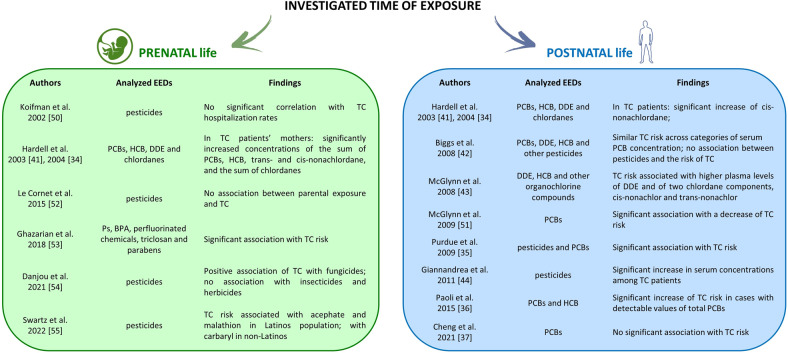
Studies that investigated the impact of prenatal and postnatal EEDs exposure on testicular cancer. EEDs environmental endocrine disruptors, TC testicular cancer, PCB polychlorinated biphenyls, HCB hexachlorobenzene, DDE dichloro-diphenyl-dichloroethylene, Ps phthalates, BPA bisphenol A

Unlike the animal models in which it has been observed several harmful consequences of EEDs on male reproductive health [48, 49], in humans, current evidence is controversial (Fig. 1).

In 2002 Koifman et al. compared the total amount of pesticide sales in 1985 and health data in a subgroup of Brazilian adolescents, finding a non-statistically significant correlation with TC hospitalization rates for the two age groups analyzed [50]. However, they did not provide information on the exact number of patients, pesticides used, or the area under analysis.

On the contrary, investigating both prenatal and postnatal PCB exposure in 61 Swedish TC patients and 44 cases’ mothers vs. their respective controls, Hardell et al. found that cases showed a significant increase of cis-nonachlordane, whereas TC patients’ mothers exhibited significantly increased concentrations of the sum of PCBs, HCB, trans- and cis-nonachlordane, and the sum of chlordanes, strengthening the putative role of fetal PCB exposure in the etiology of TC [34, 41].

In agreement with Koifman et al., not even Biggs et al. observed an association between adult exposure to organochlorine pesticides and the risk of TC [42]. However, it should be noted that this epidemiological study was carried out in blood samples of 272 patients diagnosed with TC after the first course of treatment; consequentially, a possible influence of the disease and/or treatment on the blood dosage of organochlorine pesticide levels cannot be excluded.

Then, analyzing prediagnostic serum samples of US soldiers, McGlynn et al. found that increased exposure to p,p’-DDE could be associated with the risk of both seminomatous and non-seminomatous TC (seminoma: OR = 1.91, 95% CI = 1.22–2.99, *p* = 0.0008; non-seminoma: OR = 1.63, 95% CI = 1.10–2.42, *p* = 0.0044). In contrast, exposure to chlordane compounds and metabolites could only be associated with the risk of seminoma (OR = 1.64, 95% CI = 1.04–2.60, *p* = 0.048). This result suggests an association between pesticide exposure in early life and the risk of TC in young men [43]. Nevertheless, the same authors reported a negative association between postnatal PCBs exposure and a decrease in TC risk in the same cohort, not supporting the hypothesis that PCB exposure increases the risk of this tumor [51].

In agreement with McGlynn et al. [43], Purdue et al. provided additional evidence that a prediagnostic serum level of p,p’-DDE was higher in TC subjects compared to matched controls in a cohort study of Norwegian individuals. Furthermore, the authors highlighted that serum concentrations of oxychlordane, *trans*-nonachlor compounds, and some PCB congeners were higher in serum of TC subjects. Noteworthy, different PCB congeners levels were lower in TC cases [35].

The putative association between TC and environmental exposure to organochlorine pesticides, including p,p’-DDE, and HCB, was also investigated in a hospital-based case-control study of 50 patients affected by TC vs. 48 controls. In this study, the authors reported a significant increase in serum concentrations of these chemicals among TC cases, supporting the hypothesis of a causal link between environmental exposure to pesticides and the etiology of TC [44].

Subsequently, in a large cohort of North European men, Le Cornet et al. discovered no association between parental exposure to pesticides and TC [52]. However, in this study the supposed exposure in the prenatal period was indirectly estimated through the parental occupations at the time; exposures with longer latency periods may have been missed, reducing accuracy of the estimations.

In an epidemiological study carried out in 125 Italian TC patients vs. 103 controls, Paoli et al. reported a statistically significant increase in TC risk in cases with detectable values of total polychlorinated organic compounds (14.4% in TC patients vs. 1.0% in controls, *p* < 0.001), suggesting that parents’ occupation and serum concentration of HCB and PCBs could represent a risk factor for TC [36].

Later, to assess any association with the onset of TC in the offspring, Ghazarian et al. examined the maternal use of personal care products during pregnancy and breastfeeding on a wide caseload of 527 TC cases’ mothers and 562 mothers of controls [53]. The authors found a significantly increased risk of TC associated with frequent maternal exposure to face lotion containing different EEDs, such as Ps and parabens (OR = 1.42, 95% CI = 1.08–1.86, *p* = 0.01).

Recently, evaluating serum levels of 56 PCBs congeners in 308 TC cases vs. 323 controls, Cheng et al. did not find an overall association between total PCBs and tumor risk [37]. However, when PCB congeners were classified into functional groups as proposed by Wolff et al. [33], the authors found an increased TC risk associated with higher serum levels of estrogenic PCBs group 1 (OR = 2.5, 95% CI = 1.3–4.7, *p* < 0.05).

In a French case-control study of 304 TC cases and 274 controls, the domestic use of the three major types of pesticides (insecticides, herbicides, and fungicides) was investigated during early periods of development, and a positive association was found between fungicides and risk of adult testicular germ cell tumors (OR = 1.73, CI = 1.04–2.87) and non-seminoma (OR = 2.44, CI = 1.26–4.74), while no association was detected for domestic use of insecticides and herbicides. Nevertheless, relying on self-reported information, this study did not provide detailed information about the quantity of pesticides applied or the specific active ingredients used [54].

Finally, evaluating the relationship between fetal exposure to agricultural pesticides and TC risk among 381 adolescents from California, Swartz et al. observed a difference in risk based on ethnicity: in detail, the Latino population displayed an increased TC risk associated with prenatal residential proximity to acephate (OR 1.30; 95% CI: 1.08–1.57) and malathion (OR 1.19; 95% CI: 1.01–1.39), non-Latinos with carbaryl (OR = 1.14, 95% CI: 1.01–1.28). This observation could explain a significant proportion of TC cases diagnosed in California, especially among Latinos born in areas where pesticides have been used. However, pesticide exposure after birth and parental occupation, which could impact TC risk, has not been assessed in this study [55].

## Conclusions

The growing interest in EEDs in recent years has led to a deeper understanding of how these substances interfere with hormone balance and potentially toxic effects on human health. In particular, one of the most discussed topics concerns the effects of pre-and postnatal exposure on the reproductive system in young and adult men. While most human epidemiological studies point toward an association between EEDs exposure and male reproductive system disorders, such as infertility and poor semen quality [36, 56–58], a causal relationship has yet to be established.

Similarly, the alleged association with TC is also debated, and epidemiological data report conflicting conclusions. However, most of the studies currently available in the literature suggest that EEDs exposure may play a pivotal role in TC development, although the molecular mechanisms are not yet fully understood. Several factors, such as the lack of long-term studies on a wide caseload, the gap between the time of EEDs exposure and clinical manifestations, the complex mode of action of these substances, the individual genetic background, and the absence of standardized clinical protocols, make it difficult to define a possible causal link. Indeed, due to the co-exposure to multiple factors and to a mixture on EEDs, the difficulty in understanding the effects of a single EED also contributes to making this topic intricate. Thus, further studies will be needed to investigate the pathophysiological role of EEDs in testicular dysgenesis and in the development of TC to confirm this supposed relationship.
